# Implementing an Electronic Health Record–Integrated Pediatric Primary Care Sleep Screener

**DOI:** 10.1001/jamanetworkopen.2025.25346

**Published:** 2025-08-05

**Authors:** Ariel A. Williamson, Maura Powell, Anthony Luberti, Gregory Lawton, Jungwon Min, Jesse Dudley, Joe Wu, Spandana Makeneni, Gabrielle DiFiore, Ekaterina Nekrasova, Mary Kate Kelly, Angela Rapposelli, James Massey, Eberechukwu A. Uwah, Ignacio E. Tapia, Alexander G. Fiks

**Affiliations:** 1 Children’s Hospital of Philadelphia, Philadelphia, Pennsylvania; 2 University of Pennsylvania, Perelman School of Medicine, Philadelphia; 3 The Ballmer Institute for Children’s Behavioral Health, University of Oregon, Portland; 4 University of Pennsylvania, School of Nursing, Philadelphia; 5 Miller School of Medicine, University of Miami, Miami, Florida

## Abstract

**Importance:**

Sleep is crucial for healthy growth, academic success, executive functioning, and mental health. However, sleep is not consistently and rigorously addressed in pediatric primary care.

**Objective:**

To describe the development and reach, adoption, implementation, effectiveness, and maintenance of a well-child visit, electronic sleep screener with educational resources in a large primary care network.

**Design, Setting, and Participants:**

In this case-control study of primary care practices in Pennsylvania and New Jersey, retrospective, observational electronic health records and implementation data were drawn for preimplementation (November 1, 2018, to September 30, 2019), phased-scaling (October 1, 2019, to June 30, 2021), implementation (July 1, 2021, to June 30, 2022), and maintenance (July 1, 2022, to June 30, 2023) periods. Multivariate logistic regression examined the effectiveness by comparing implementation vs preimplementation rates of sleep disorder diagnosis, polysomnogram orders, and sleep-related referrals. Patients were seen for a well-child visit during the preimplementation and implementation periods, without exclusions. Data were analyzed from October 10, 2023, to May 2, 2025.

**Exposure:**

An age-based, electronic sleep screener assessing infant bed sharing, frequent snoring (≥3 nights/week), perceived sleep problems, insufficient sleep duration, and adolescent daytime sleepiness.

**Main Outcomes and Measures:**

The Reach, Effectiveness, Adoption, Implementation, and Maintenance framework guided the outcomes including sleep screener use, results, and primary care clinician (PCC)–rendered sleep disorder diagnosis, polysomnogram orders, and sleep-related referrals at the well-child visit.

**Results:**

A total of 409 217 well-child visits for 288 307 unique patients aged 18 years or younger (51.2% male; 49.9% White non-Hispanic or Latine) were included in the preimplementation and implementation periods. During implementation, 204 872 unique patients in 31 practices completed the screener, with adoption in 89.5% of all well-child visits. Overall, 9.7% of patients endorsed frequent snoring, 12.2% sleep problems, and 34.4% insufficient sleep. Infant bed sharing was endorsed in 6.5% of infants, whereas 14.7% of adolescents endorsed daytime sleepiness. Compared with the preimplementation period, at well-child visits with a completed sleep screener, PCCs were significantly more likely to render a sleep disorder diagnosis (odds ratio, 1.64 [95% CI, 1.56-1.73]), order a polysomnogram (odds ratio, 2.67 [95% CI, 2.32-3.20]), and refer to sleep (odds ratio, 6.48 [95% CI, 5.03-8.34]) or otolaryngology (odds ratio, 4.46 [95% CI, 3.95-5.02]) clinics. Minimal adaptations occurred during implementation, and adoption was high and persistent (92.5% of well-child visits) during the maintenance period.

**Conclusions and Relevance:**

In this case-control study, a brief, electronic well-child visit sleep screener was widely adopted and maintained in a sociodemographically diverse primary care network and was associated with increased recognition and management of sleep problems.

## Key Points

**Question:**

Is integrating an electronic sleep screening tool with educational resources at well-child visits feasible, scalable, sustainable, and associated with enhanced sleep problem identification and management in primary care?

**Findings:**

This case-control study with 409 217 well-child visits among 288 307 unique patients demonstrated high reach, adoption, and maintenance of an electronic, well-child visit sleep screening tool with educational resources in a large, sociodemographically diverse pediatric primary care network, with evidence of enhanced sleep problem identification and management.

**Meaning:**

The findings suggest that the sleep screener provides a feasible and scalable approach to implement American Academy of Pediatrics guidance on screening for and promoting sleep health as part of routine well-child care.

## Teaser

This case-control study of primary care practices in Pennsylvania and New Jersey describes the development, adoption, and outcomes of an electronic sleep screener with educational resources for use in well-child visits.

## Introduction

Healthy sleep is vital during childhood. Poor sleep health (eg, short duration) and untreated medical (eg, sleep-disordered breathing [SDB]) and behavioral (eg, insomnia) sleep disorders in childhood are associated with obesity,^1^ academic problems,^2,3^ and mental health concerns, including suicidal ideation, suicide attempts, and suicide completion.^4^ Despite sleep’s importance for optimal development, sleep problem symptoms are commonly underidentified and undertreated in outpatient settings, including primary care.^5^

The American Academy of Pediatrics (AAP) recommends assessing for and promoting sleep health (eg, sufficient duration, regular bedtime).^6^ Like sleep-focused organizations, the AAP also recommends well-child visit screenings for habitual snoring (≥3 nights/week), a common obstructive sleep apnea symptom.^7^ Yet primary care clinicians (PCCs) lack standardized tools that feasibly, efficiently, and effectively identify and manage sleep problems while also minimizing rather than contributing to screening burden. A 5-domain primary care screener was associated with increased identification of sleep problems,^8^ but it was implemented via pencil and paper for 195 children at 1 clinic, limiting generalizability. In 1 health care system, a brief electronic infant sleep questionnaire^9^ and an SDB screener with clinical decision support were feasibly implemented during well-child visits^10^; however, other sleep domains were not evaluated across ages or in more than 5 practices.

To address the lack of an efficient and effective tool to support PCCs in identifying and managing sleep problems, we developed and implemented a brief, electronic health record (EHR)–integrated well-child visit sleep screener with educational resources. The purpose of this study was to describe the developmment of the sleep screener and evaluate its implementation for and association with sleep problem recognition and management in a large primary care network using the Reach, Effectiveness, Adoption, Implementation, Maintenance framework.^11,12^ We hypothesized that the screener would reach 80% or more of unique patients and be adopted in 80% or more of well-child visit encounters during its first year of network-wide implementation. We also hypothesized that the implementation of the screener would be associated with increased sleep disorder diagnosis and management (orders or referrals) compared with a preimplementation period.

## Methods

### Design and Setting

Through the Possibilities Project, the Children’s Hospital of Philadelphia (CHOP) primary care innovation program, we conducted a retrospective, observational case-control study in the CHOP primary care network of 31 practices in Pennsylvania and New Jersey. Twenty-seven practices were in suburban or rural settings, and 4 were in large, metropolitan settings. Yearly, the network serves more than 200 000 patients aged 18 years or younger.

The Figure defines the 4 study periods, which are (1) the preimplementation period (November 1, 2018, to September 30, 2019), when no screening occurred and when there was no overlap with the coronavirus pandemic onset (March 2020), which initially impacted clinical care; (2) the phased-scaling period (October 1, 2019, to June 30, 2021), when the screener was iteratively refined and implemented in selected clinics; (3) the network-wide implementation period (July 1, 2021, to June 30, 2022), when the screener was available in all 31 practices; and (4) the maintenance period (July 1, 2022, to July 1, 2023), a 12-month period following implementation.

**Figure.**
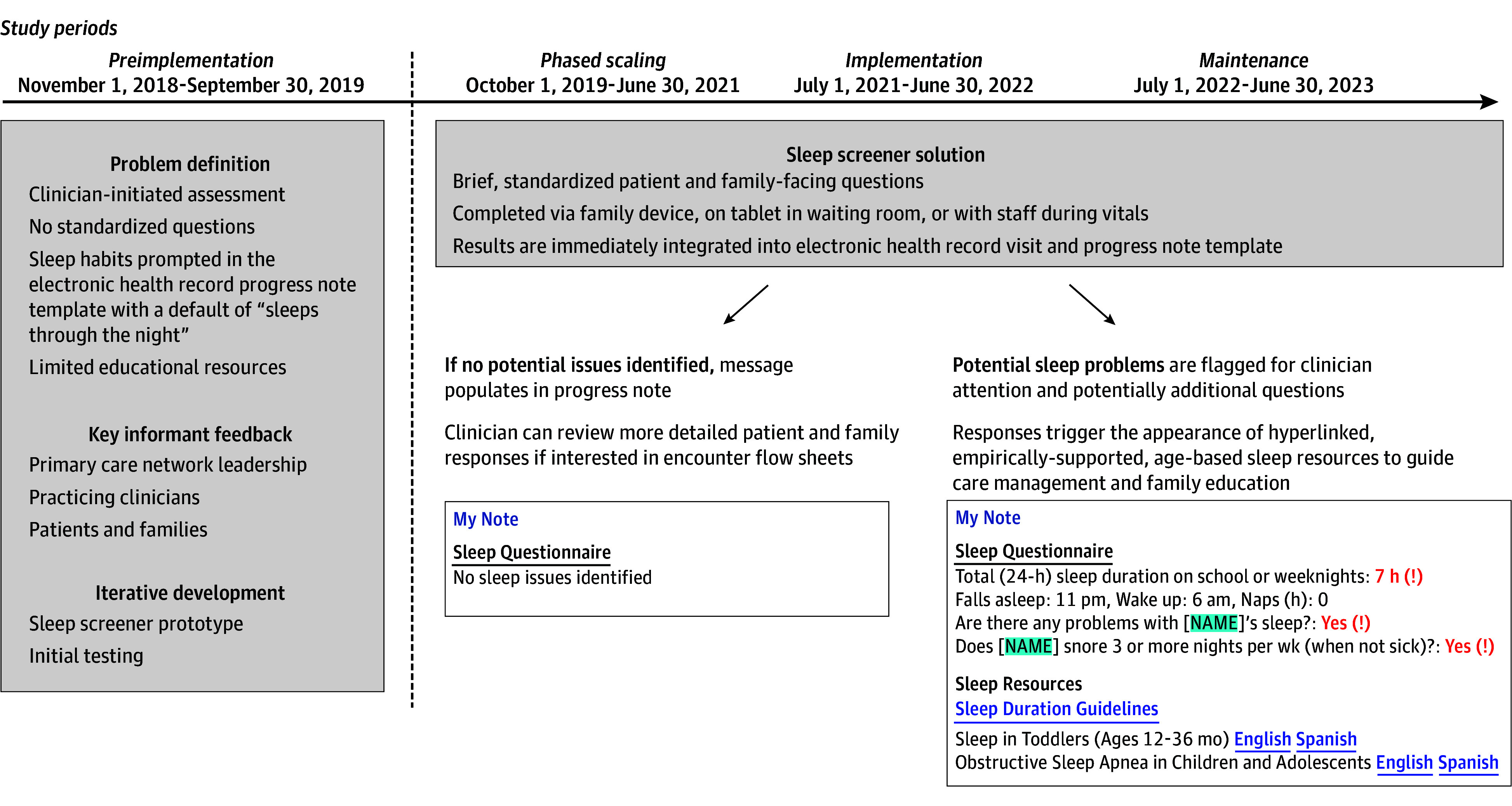
Sleep Screener Development and Implementation Timeline, Workflow, and Electronic Health Record Integration Summary of the sleep screener completion flow during well-child visits and its development over the 4 study periods, including (1) the preimplementation period, when no screening occurred and there was no overlap with coronavirus pandemic onset (March 2020); (2) the phased-scaling period, when the screener was iteratively refined and implemented in selected clinics; (3) the network-wide implementation period, when the screener was available in all 31 practices; and (4) the maintenance period, a 12-month period following implementation.

### Sleep Screener Development

During the preimplementation period (Figure), a pediatric sleep expert (A.A.W.), practicing PCCs (A.L., G.L., and A.G.F.), and a quality improvement expert (M.P.) collaboratively defined the scope of the problem that a well-child visit screener could address. We found that there was no standardized approach for identifying or documenting sleep problems, including the AAP-recommended snoring^7^ and sudden infant death syndrome risk^13^ screenings. Previously at CHOP, only a drop-down list of sleep characteristics in the well-child visit progress note template was available, with “sleeps through the night” as the default (Figure). Based on PCC feedback, the screener was designed for efficient previsit completion by patients or families electronically at home or with staff during vital signs assessment, instead of prompting PCCs to screen during the visit. Network leaders prioritized a brief screener and efficient EHR integration, given short visits with many existing screeners (eg, child development, adolescent depression). We also found that patient and family sleep education handouts lacked tailoring to different ages and sleep problems.

We then identified key sleep domains and items by age (Table 1; bed sharing [infants only], habitual snoring, total [24-hour] sleep duration, sleep problems, daytime sleepiness [adolescents only]).^1,2,3,7,8,10,13,14,15,16,17,18,19,20,21,22,23,24,25,26,27,28,29,30,31,32^ Domains were based on AAP (sudden infant death syndrome,^13^ SDB^7^) and American Academy of Sleep Medicine (duration^26^) guidance; research specifically linking sleep duration,^1^ problems,^21^ and daytime sleepiness^31^ to child outcomes; and population-level sleep surveillance (Centers for Disease Control and Prevention^14^; National Sleep Foundation^28^). Age-based items were adapted from population-level surveillance and validated questionnaires to ensure brevity and conform with the screener purpose of eliciting initial information that PCCs could use for further conversation and management. For simplicity and efficient EHR integration, a “yes” response to yes or no items reflected “abnormal” endorsement (Figure).

**Table 1.** Identified Key Sleep Domains, Rationale for Inclusion, Screening Questions by Age, and Relevant Sources for Question Wording^a^

Key sleep domain	Rationale	Question wording and responses by age^b^	Sources for question wording
Sleep position	Aligns with AAP safe sleep guidelines^13^ Aligns with Centers for Disease Control maternal or child health surveillance^14^	0-5 mo and 6-11 mo: Does [child’s name] sleep with you or another adult? (yes or no) All other age groups: not included due to older age.	Newborn Sleep Safety Survey^15^ Pregnancy Risk Monitoring Assessment System^14^
Sleep disordered breathing	Aligns with AAP sleep-disordered breathing recommendations^7^ Even mild snoring is associated with impaired child functioning^3^	0-5 mo: Not included due to young age. ≥6 mo: Does [child’s name] snore 3 or more nights per week (when not sick)? (yes or no)	AAP guidelines^7^ Pediatric Sleep Questionnaire^16^ BEARS sleep screening instrument^8^ CHICA–Obstructive Sleep Apnea module^10^
Perceived sleep problem	Strongly associated with other behavioral sleep problems (eg, difficulty falling asleep, frequent night awakenings)^17^ Strongly associated with physical and behavioral health concerns^18,19^ Part of diagnostic criteria for pediatric insomnia^20^ Represents patients or families that may benefit from behavioral sleep intervention^21^	0-5 mo: Not included due to young age and normative night awakenings in infancy^22^ ≥6 mo: Are there any problems with [child’s name]’s sleep? (yes or no)	Brief Infant/Child Sleep Questionnaire^23^ BEARS sleep screening instrument^8^ PROMIS Sleep Disturbance measure^24^
Sleep duration	Sleep duration is the only sleep health domain^25^ with specific, age-based national recommendations^26^ Aligns with Centers for Disease Control sleep surveillance^27^ Strongly associated with multiple aspects of child development and functioning^1^	0-5 mo and 6-11 mo: Not included due to young age and significant variability in infant sleep duration^22^ ≥12 mo: Duration calculated from the following: On school or weeknights, [child’s name] usually falls asleep around _____. (eg, your child goes to bed around 7:30 pm but falls asleep around 8:00 pm.) (7 pm, 7:30 pm, 8 pm, 8:30 pm, 9 pm, 9:30 pm, 10 pm, 10:30 pm, 11 pm, 11:30 pm, midnight, 12:30 am, 1 am or later) On school or weekdays, [child’s name] usually wakes up around _____. (4 am, 4:30 am, 5 am, 5:30 am, 6 am, 6:30 am, 7 am, 7:30 am, 8 am, 8:30 am, 9 am, 9:30 am, 10 am or later) On school or weekdays, [child’s name] usually naps for a total of _____ hours. (0, 0.5, 1. 1,5, 2, 2.5, 3, 3.5, 4 or more) Insufficient sleep identified according to age-based 24-h guidelines:^26^ 12-23 mo: <11 h 3-5 y: <10 h 6-12 y: <9 h ≥13 y: <8 h	National Sleep Foundation Sleep in America Poll^28^ American Academy of Sleep Medicine guidelines^26^ Youth Risk Behavior Survey^27^ Children’s Sleep Habits Questionnaire^29^
Daytime sleepiness	Associated with adolescent functioning, including driving risk^2,30^ Increased prevalence in adolescence^31^	All younger ages: not included due to young age and/or need for screener brevity ≥13 y: Does [child’s name] ever fall asleep in school? (yes or no)	PROMIS sleep-related impairment measure^24^ Epworth Sleepiness Scale for Children^32^

Abbreviations: AAP, American Academy of Pediatrics; BEARS, bedtime problems, excessive daytime sleepiness; awakening during night; regular and duration of sleep; sleep-disordered breathing; CHICA, Child Health Improvement through Computer Automation; PROMIS, Patient-Reported Outcomes Measurement Information System.

^a^ Well-child visits by age: 0 to 5 months = newborn and 1-, 2-, and 4-month visits; 6 to 11 months = 6- and 9-month visits; 12 to 23 months = 12-, 15-, and 18-month visits; 24 to 35 months = 24- and 30-month visits; 3 to 5 years = 3-year, 42-month, 4-year, and 5-year visits; 6 to 12 years = 6-, 7-, 8-, 9-, 10-, 11-, and 12-year visits; 13 years or older = 13-, 14-, 15-, 16-, 17-, and 18-year visits.

^b^ See eTable 4 in Supplement 1 for original item wording used during implementation.

We then developed prototypes for the appearance of the screener and results in the patient and family-facing portal and EHR. One clinician (G.L.) piloted the prototype with patients at 1 practice for further refinement. Other sleep clinicians, PCCs, and researchers provided feedback and updated patient and family education handouts with age- and evidence-based sleep health guidance,^33^ including the recommended duration^26^ and information for the hospital’s sleep and otolaryngology clinics. Handouts, appearing as progress note hyperlinks, were tailored to screener results to guide PCC management. For example, a 30-month-old with endorsed snoring and sleep problem items would generate hyperlinked resources for SDB psychoeducation and healthy sleep guidance for toddlers (Figure). We further piloted and refined the screener and resources during the phased-scaling period for all well-child visits at the practice where the prototype was tested, with ongoing practice and patient and family feedback. The screener was developed using standard functionality in Epic to facilitate scaling within and beyond the CHOP health system.

### Assessments

Table 2 shows operationalized outcomes organized according to the Reach, Effectiveness, Adoption, Implementation, Maintenance framework.^11,12,34^ Retrospective EHR data were used to assess screener use among unique patients (reach) and at the well-child visit encounter and practice levels (adoption) during implementation. We examined effectiveness by comparing rates of sleep-related identification (PCC-rendered sleep disorder diagnosis) or management (polysomnogram [PSG] order or referrals to the hospital’s sleep and/or otolaryngology clinics) on the date of the well-child visit from preimplementation to postimplementation periods. We also examined the proportion of patients with PCC-rendered sleep disorder identification and management activities at the well-child visit that had sleep problems endorsed on the same-day screener. These outcomes represent the desired effects of a screener designed to detect potential sleep problems, similar to other primary care screening research^34^ and different from research evaluating a sleep intervention, which would assess for changes in sleep problems. To assess implementation, we documented adaptations made to the screener items and delivery methods during phased scaling and implementation. To assess maintenance, we used EHR data and implementation team notes from the maintenance period (Table 2). There were no participant exclusion criteria. The CHOP institutional review board reviewed and approved this study with a waiver of Health Insurance Portability and Accountability Act authorization and consent. This case-control study followed the Strengthening the Reporting of Observational Studies in Epidemiology (STROBE) reporting guideline.

**Table 2.** Outcomes Organized by RE-AIM Dimensions

RE-AIM dimensions	Study definition	Study measurement
Reach: the proportion, number reached, and characteristics of patients exposed to the innovation	Total number of unique patients with a completed sleep screener seen during implementation, across practices Absolute proportion of unique patients with a completed sleep screener of all unique patients during implementation, across practices	EHR-identified completion (coded as 1) or noncompletion (coded as 0) of sleep screener at unique patient level
Effectiveness: the impact of the innovation on relevant outcomes	Comparison of rates of sleep diagnosis and management at well-child visits during the preimplementation vs implementation periods Absolute proportion of patients with a sleep screener item endorsed and clinically relevant outcomes (sleep diagnosis and management) on the date of the well-child visit, of those that received well-child visit sleep diagnosis and management: PCC-rendered sleep diagnosis Polysomnogram ordered Sleep clinic referral Otolaryngology referral	EHR-identified outcomes at the well-child visit: *International Statistical Classification of Diseases and Related Health Problems, Tenth Revision* sleep disorder diagnosis (coded as 1) rendered by PCC PCC-ordered polysomnogram (coded as 1) PCC referral to sleep clinic (coded as 1) or otolaryngology (coded as 1) EHR-identified presence (coded as 1) of: Endorsement of snoring, sleep problems, insufficient sleep, or daytime sleepiness at visit in which PCC rendered a sleep disorder diagnosis, ordered a PSG, or made a referral to sleep clinic or otolaryngology, of those with PCC-rendered sleep diagnosis and management at the well-child visit. Endorsement of any sleep screener item at visit in which PCC rendered a sleep disorder diagnosis, ordered a PSG, or made a referral to sleep clinic or otolaryngology, of those with PCC-rendered sleep diagnosis and management at the well-child visit^a^
Adoption: the proportion or number of those willing to implement the innovation	Absolute proportion of sleep screeners completed at a well-child visit of all well-child visits conducted during implementation, across practices Range of practice-level proportions for completed sleep screeners at a well-child visit of all well-child visits conducted during implementation	EHR-identified completion (coded as 1) or noncompletion (coded as 0) of sleep screener at the encounter level, overall and by practice
Implementation: the fidelity and adaptations made to the innovation during delivery	Adaptations made to the screener questions and delivery methods in response to clinician and/or practice feedback during phased scaling and implementation periods	Study team documentation of clinician and/or practice feedback and subsequent adaptations
Maintenance: the extent to which the innovation is maintained as part of usual practice in the medium to long term	Adoption outcomes (described previously) during maintenance, across practices Implementation outcomes (described previously) during maintenance, across practices	EHR-identified completion (coded as 1) or noncompletion (coded as 0) of sleep screener at the encounter level, overall and by practice Study team documentation of feedback and subsequent adaptations

Abbreviations: EHR, electronic health record; PCC, primary care clinician; PSG, polysomnogram; RE-AIM, Reach, Effectiveness, Adoption, Implementation, Maintenance framework.

^a^ Any endorsement indicates any screener item endorsed of the 4 listed (snoring, sleep problem, insufficient sleep, daytime sleepiness); sharing a sleep space with an adult was not examined given that the sleep diagnosis and management activities are not relevant for this concern. Study periods: preimplementation (11/1/2018-9/30/2019); phased scaling (10/1/2019-6/30/2021); implementation (7/1/2021-6/30/2022); maintenance (7/1/2022-6/30/2023).

### Sociodemographic Information

We extracted EHR data for well-child visit patients seen during the preimplementation and implementation periods (120 910 patients were seen in both periods) to characterize the sample and include as covariates in effectiveness analyses, given research showing sociodemographic variation and disparities in child sleep.^35,36,37^ Data included child age, sex assigned at birth, race and ethnicity (reflecting sociopolitical and not genetic or biological constructs),^38^ insurance, and the Childhood Opportunity Index (COI),^39^ to reflect family and neighborhood socioeconomic status (SES), respectively (eTable 1 in Supplement 1). Racial and ethnic groups included African American or Black, Asian, Hispanic or Latine, White non-Hispanic or Latine, multiple races, and other. The groups included in the “other” category were American Indian, Alaska native, Eskimo, or “other” (ie, some other race not identified in the list provided). Additional information about “other” and “multiple races” is not available in our dataset and as such cannot be accessed. Race and ethnicity were entered by patients, a family member, or a staff member upon patient registration into hospital-defined categories that appear in the EHR. These data were drawn from the EHR. Race and ethnicity data were collected to characterize the sample for generalizability purposes and in light of well-documented disparities by race and ethnicity in pediatric sleep health.

### Statistical Analysis

We used Stata, version 16 (StataCorp LLC) and SAS, version 9.4 (SAS Institute Inc) for analyses with *P* < .05 for 2-sided tests of statistical significance. Statistical analysis was conducted from October 10, 2023, to May 2, 2025. Descriptive statistics characterized patient sociodemographic characteristics, reach, adoption, and maintenance. The χ^2^ test was used to examine any sociodemographic differences among patients seen during the preimplementation and implementation periods, with phi (φ) and the Cramer *V* evaluating the strength of any differences. For effectiveness, mixed-effects logistic regression models with covariates (child age, sex, race, ethnicity, insurance coverage, and COI) compared the likelihood of the identification and management of a sleep disorder at a well-child visit during the implementation and preimplementation periods, accounting for clustering by patients and clinics. Descriptive statistics characterized the proportion of unique patients with sleep screener items endorsed and a sleep disorder diagnosis and management at the well-child visit, of those with diagnosis and management activities at the well-child visit. For patients with multiple visits in 1 period, data from their first well-child visit in each period were used. Since less than 1.0% of the sociodemographic data were missing in our analytic dataset, we performed a complete case analysis by assuming missingness occurred completely at random. Although 7.0% of patients had an unidentifiable residential address to define COI (eg, a PO Box typograpical error), sensitivity analyses conducted without COI adjustment yielded trivial differences in estimates.

## Results

### Preliminary Analyses

A total of 409 217 well-child visits for 288 307 unique patients aged 18 years or younger (51.2% male; 5.0% African American or Black, 23.7% Asian, 8.7% Hispanic or Latine, 49.9% White non-Hispanic or Latine, 3.1% multiple races, and 8.9% other; 30% Medicaid insured and 70% commercially insured) seen in the preimplementation and implementation periods were included (120 910 patients seen in both periods). eTable 2 in Supplement 1 provides sociodemographic characteristics, which align with network estimates.^40^ The few statistically significant differences between periods were negligible based on φ and Cramer *V*.^41^

During implementation and across age groups, 9.7% of patients endorsed snoring, 12.2% sleep problems, and 34.4% insufficient sleep, and 6.5% of infants had reported bed sharing. Daytime sleepiness was endorsed in 14.7% of adolescents. Table 3 shows sleep screener item endorsement by age as well as sleep problem estimates from previous research.^9,14,15,20,22,27,28,31,36,42^

**Table 3.** Sleep Screener Endorsement and Effectiveness Outcomes and Among Those With Sleep Management on Day of Well-Child Visit During the Implementation Period (N = 204 345)

Child age (No.)^a^	Sleep screener item endorsement, No. (%)					
	Sleeping with an adult	Snoring ≥3 nights/wk	Sleep problem	Insufficient sleep	Daytime sleepiness	Any item endorsement
0-5 mo (n = 16 730)	841 (5.0)	NA	NA	NA	NA	841 (5.0)
6-11 mo (n = 5476)	606 (11.1)	504 (9.2)	508 (9.3)	NA	NA	1349 (24.6)
12-23 mo (n = 11 604)	NA	1253 (10.8)	1074 (9.3)	1498 (12.9)	NA	2988 (25.8)
24-35 mo (n = 11 751)	NA	1318 (11.2)	1286 (10.9)	2193 (18.7)	NA	3652 (31.1)
3-5 y (n = 34 699)	NA	3351 (9.7)	4007 (11.6)	6339 (18.3)	NA	10 423 (30.0)
6-12 y (n = 73 951)	NA	7328 (9.9)	8286 (11.2)	26 398 (35.7)	NA	33 066 (44.7)
≥13 y (n = 50 661)	NA	4410 (8.7)	7780 (15.4)	26 468 (52.3)	7432 (14.7)	30 769 (60.7)
Across all ages	1447 (6.5)	18 164 (9.7)	22 941 (12.2)	62 896 (34.4)	7432 (14.7)	83 088 (40.6)
Estimates from prior research	29-60^14,15^	6-17^42^	7-30^9,20,22,36^	30-70^27,28^	20-47^31^	NA
Well-child visit clinical sleep management for those with an endorsed sleep problem, No./total No. (%)						
Any sleep disorder diagnosis	NA^b^	2716/5153 (52.7)^c^	2614/5153 (50.7)^d^	2761/5084 (54.3)^e^	382/1151 (33.2)^f^	4625/5153 (89.8)^g^
PSG ordered	NA	372/443 (84.0)	202/443 (45.6)	245/440 (55.7)	31/93 (33.3)	419/443 (94.6)
Sleep clinic referral	NA	223/548 (40.7)	423/548 (77.2)	373/541 (69.0)	41/98 (41.8)	520/548 (94.9)
Otolaryngology referral	NA	288/440 (65.5)	134/440 (30.5)	143/431 (33.2)	11/34 (32.4)	332/440 (75.5)

Abbreviations: NA, not applicable; PCC, primary care clinician; PSG, polysomnogram.

^a^ Sample size varies for age-based questions as follows: sleeping with an adult (n = 22 206), snoring (n = 188 142), sleep problem (n = 188 142), insufficient sleep (n = 182 666), and daytime sleepiness (n = 50 661).

^b^ Sharing a sleep space with an adult was not examined given that the sleep diagnosis and management activities listed are not relevant for this concern.

^c^ For this column, the numerator is number of patients aged 6 months or older with snoring endorsed on sleep screener and PCC-rendered diagnosis or management at well-child visit; the denominator is number of all patients aged 6 months or older with PCC-rendered diagnosis or management at well-child visit.

^d^ For this column, the numerator is number of patients aged 6 months or older with a sleep problem endorsed on sleep screener and PCC-rendered diagnosis or management at well-child visit; the denominator is number of all patients aged 6 months or older with PCC-rendered diagnosis or management at well-child visit.

^e^ For this column, the numerator is number of patients aged 12 months or older with insufficient sleep endorsed on sleep screener and PCC-rendered diagnosis or management at well-child visit; the denominator is number of all patients aged 12 months or older with PCC-rendered diagnosis or management at well-child visit.

^f^ For this column, the numerator is number of patients aged 13 years or older with daytime sleepiness endorsed on sleep screener and PCC-rendered diagnosis or management at well-child visit; the denominator is number of all patients aged 13 years or older with PCC-rendered diagnosis or management at well-child visit.

^g^ For this column, the numerator is number of patients aged 6 months or older with any of the 4 screener items listed (snoring, sleep problem, insufficient sleep, daytime sleepiness) endorsed on the sleep screener and PCC-rendered diagnosis or management at the well-child visit; the denominator is number of all patients aged 6 months or older with any PCC-rendered diagnosis or management at well-child visit.

### Implementation

During phased scaling, we approached an increasing number of network practices to implement the screener while monitoring adoption via EHR data. For each site, we provided a 30-minute virtual education session with PowerPoint slides to orient PCCs, medical assistants, and other staff members to the screener items, workflow, and resources. In response to PCC and patient and family feedback gathered at practice meetings, we added patient and family handouts with information about melatonin and PSGs. The screener and educational resources were also translated into Spanish. No further adaptations were made during implementation.

### Reach and Adoption

During implementation, the sleep screener was completed by 204 872 unique patients, or 89.3% of unique well-child visit patients (n = 229 489), suggesting broad reach (≥80%). At the encounter level, adoption rates were high (≥80%), with the screener completed in 303 716 of 339 274 well-child visit encounters (89.5%). Practice level (eFigure in Supplement 1) rates of well-child visit encounter adoption varied from 71% to 99%, with nearly all (n = 29) practices adopting the screener in 80% or more of well-child visits.

### Effectiveness

PCC-rendered sleep disorder diagnoses (8634 of 409 217 well-child visits [2.1%]), PSG orders (631 of 409 217 well-child visits [0.2%]), and sleep-related referrals (645 of 409 217 well-child visits [0.2%]) were relatively low across the preimplementation and implementation periods (Table 4). During the preimplementation period with 204 345 well-child visits, there were 3446 visits (1.7%) in which children received a PCC-rendered sleep disorder diagnosis (eTable 3 in Supplement 1 shows specific diagnosis prevalence), 187 visits (0.1%) in which children received a PSG order, 94 visits (<0.001%) in which children were referred to the sleep clinic, and 421 visits (0.2%) in which children were referred to otolaryngology. During the implementation period with 204 872 well-child visits, however, there were 5188 visits (2.5%) in which children with a completed sleep screener received a PCC-rendered diagnosis, 444 visits (0.2%) in which children received a PSG order, 551 visits (0.3%) in which children were referred to the sleep clinic, and 1717 visits (0.8%) in which children were referred to otolaryngology. Compared with the preimplementation period, children during the implementation period were 64% more likely to receive a PCC-rendered sleep disorder diagnosis at the well-child visit (odds ratio, 1.64 [95% CI, 1.56-1.73]; *P* < .001). PCCs were also twice as likely as during the preimplementation period to order a PSG (odds ratio, 2.67 [95% CI, 2.22-3.20]; *P* < .001) and much more likely to make a referral to sleep clinic (odds ratio, 6.48 [95% CI, 5.03-8.34]; *P* < .001) or otolaryngology (odds ratio, 4.46 [95% CI, 3.95-5.02]; *P* < .001) at the well-child visit.

**Table 4.** Effectiveness: Sleep Management During the Preimplementation and Implementation Periods

Well-child visit clinical sleep management	Visits, No. (%)			OR (95% CI) for implementation vs preimplementation^a^
	All (n = 409 217)	Preimplementation, 11/1/2018-9/30/2019 (n = 204 345)	Implementation, 7/1/2021-6/30/2022 (n = 204 872)	
Any sleep disorder diagnosis	8634 (2.1)	3446 (1.7)	5188 (2.5)	1.64 (1.56-1.73)^b^
PSG ordered	631 (0.2)	187 (0.1)	444 (0.2)	2.67 (2.22-3.20)^b^
Sleep clinic referral	645 (0.2)	94 (<0.001)	551 (0.3)	6.48 (5.03-8.34)^b^
Otolaryngology referral	2138 (0.5)	421 (0.2)	1717 (0.8)	4.46 (3.95-5.02)^b^

Abbreviations: OR, odds ratio; PSG, polysomnogram.

^a^ Mixed-effects logistic regression models accounting for clustering by patients (120 910 seen in both periods) and primary care clinics (N = 31) and adjusted for child age, sex, race and ethnicity, insurance, and Childhood Opportunity Index. Sensitivity analyses conducted without the Childhood Opportunity Index adjustment showed consistent patterns of significance and effect sizes.

^b^ *P* < .05.

To further validate that children with a PCC-rendered sleep disorder diagnosis or management activities at a well-child visit during the implementation period had potential sleep problems, we examined screener endorsement in this population (Table 3). We found very high levels of sleep screener endorsement. Specifically, 89.8% (4625 of 5153) of those with a PCC-rendered sleep disorder diagnosis at the well-child visit had 1 or more endorsed items on the same-date sleep screener. Similarly, 94.6% (419 of 443) of those with PSG orders, 94.9% (520 of 548) of those with sleep clinic referrals, and 75.5% (332 of 440) of those with otolaryngology referrals made by the PCC at the well-child visit had 1 or more endorsed sleep screener items.

### Maintenance

Practice feedback resulted in 2 screener adaptations. To increase clarity, all items were revised to include the child’s name (eg, “Are there any problems with [name’s] sleep?”) rather than asking about “your baby/child/teen.” Because PCCs, patients, and families indicated that it was challenging to calculate sleep duration, we replaced the original sleep-duration item with separate fall asleep time, wake time, and nap duration items. Child sleep duration automatically calculates from these items, with a flag for insufficient duration according to age-based guidelines (Table 1; see eTable 4 in Supplement 1 for original wording).

Adoption rates remained at 80% or higher and increased during maintenance, with the screener completed in 92.9% of well-child visit encounters (329 329 of 354 515). Practice level adoption ranged from 79% to 98%, with adoption of 80% or more in 30 of 31 practices (eFigure in Supplement 1).

## Discussion

This case-control study is among the first to demonstrate the reach, effectiveness, adoption, implementation, and maintenance of a brief, patient and family-facing, EHR-integrated well-child visit sleep screener with educational resources in a large, sociodemographically diverse pediatric primary care network. The screener was adopted in 89.5% of well-child visits in its first year of implementation, with substantially higher rates of sleep problem identification than in a prior study in this network.^43^ Screener implementation was additionally associated with increased PPC-rendered sleep disorder diagnoses, PSG orders, and referrals to sleep and/or otolaryngology clinics at the time of the well-child visit compared with preimplementation rates of these activities at well-child visits. Importantly, high adoption rates overall and at the practice level were sustained and even increased during maintenance.

Several factors were likely associated with the high rates of screener adoption and maintenance. The screener and educational resources were developed to maximize efficiency, with tight integration into PCCs’ workflows and progress notes. Using electronic rather than paper-based screening and relying on patient and family-facing questions rather than clinician-initiated assessment also likely was associated with widespread, sustained adoption.^44,45^ Abnormal responses were also flagged for PCCs, streamlining assessment. Further, sleep resources were provided at the point of care, which can help standardize PCC guidance and patient and family education. As PCCs must balance many screenings and intervention demands in brief visits, our approach to optimizing EHR-integrated screeners with educational resources may support more efficient and effective care.^46^ Incorporating feedback from end users (PCCs) and screener recipients (patients and families) in the settings in which it was ultimately designed for use likely supported implementation.^47^ Although we did not systematically assess PCC, patient, and family acceptability of the screener and resources, positive reports from practices guided continued refinement.

Importantly, screener implementation was associated with increased PCC-rendered sleep disorder identification and management activities at the well-child visit—which are the desired outcomes of a screener. However, rates were still lower than 3%. Low diagnostic rates could be reasonable as symptoms reported at well-child visits may not meet diagnostic thresholds^20^ or may require further assessment (eg, PSG to diagnose obstructive sleep apnea).^7^ Although the implementation process included clinician-facing sleep education, more PCC-directed sleep resources may be needed.^5,48^ Low diagnostic rates could explain the low rates of PSG orders and sleep-related referrals, as PCCs and/or patients and families may not consider these services to be relevant if screener-endorsed symptoms are subthreshold or not of concern. Of note, most children with a PCC-rendered sleep diagnosis, PSG orders, and/or relevant referrals also had endorsed 1 or more screener items, suggesting that screener endorsement could have driven these PCC activities at the well-child visit although causality cannot be determined given the cross-sectional nature of this study.

Across ages, reported habitual snoring, sleep problems, and insufficient sleep aligned with estimates from diagnostic and/or epidemiologic research (Table 3) that at least 10% of children habitually snore,^7^ 7% to 30% experience insomnia symptoms,^9,22,49^ and 30% to 60% experience insufficient sleep.^27,28^ Although endorsing broad sleep problems (yes to “Are there any problems with [name]’s sleep?”) could reflect other sleep disorder symptoms (eg, parasomnias), research indicates endorsement is robustly correlated with insomnia symptoms (difficulty falling or staying asleep).^17,23^ Broad sleep problems were more common among children aged 6 to 12 years and those aged 13 years or older, unlike in prior studies indicating greater prevalence during early childhood.^17,20^ Our findings could be due to the global increase in pandemic-related mental health concerns among school-aged children and adolescents,^50^ given robust correlations between sleep and mental health.^18,19^ Infant bed sharing was much less prevalent compared with epidemiologic research,^14,15^ potentially due to methodological variation, as families may be less likely to disclose unsafe sleep practices when asked as part of visit questionnaires or by PCCs^51^ than when completing questionnaires anonymously. Low prevalence rates across sleep problems in our study could also be due to using single items to reflect these constructs, unlike prior research using multiple items and/or questionnaires. With input from clinicians and families, single items were chosen to minimize burden.

### Limitations

Findings should be considered in the context of study limitations. The lack of control practices limits our ability to make conclusions about causality and to account for secular and seasonal trends, especially as the preimplementation and implementation periods were not seasonally aligned. EHR limitations prevented us from quantifying rates of printed or electronically delivered patient and family educational resources. Future research should examine this and other outcomes, such as clinical efficiency, screener costs, and longer-term maintenance.^34^ Findings were also limited by a lack of validation data for screener-endorsed sleep problems, such as SDB and potential insomnia symptoms. However, screener items were adapted from validated questionnaires and population-based surveillance (Table 1). Nonetheless, future studies should evaluate screener item performance against other diagnostic and/or evaluative measures, including PSG for obstructive sleep apnea evaluation, actigraphy for sleep duration, and patient and family interviews for insomnia disorder. Despite having a large, sociodemographically diverse sample, this study was conducted in 1 network affiliated with an academic medical center and specialty sleep and otolaryngology clinics, potentially limiting generalizability. Studies in other contexts and that evaluate equity in screener implementation are needed given pediatric sleep health disparities.^35,36,37^

## Conclusions

In this case-control study of a patient and family-facing, EHR-integrated sleep screener with educational resources, we found this tool was feasible, widely adopted, and associated with improvements in sleep problem identification and management during well-child visits. The high prevalence of endorsed sleep problems underscores the urgent need for efficient and effective methods to address these concerns at the point of care. Future research should examine whether a primary care–based sleep intervention following a screening could reduce sleep problems, as the screener was designed to facilitate problem identification and initial management, not reduce sleep problems. Our successful development and implementation of the sleep screener and educational resources using a scalable approach are initial steps toward not only identifying sleep problems but also promoting sleep health and broader well-being in pediatric primary care.

## Notes

Supplement 1. **eTable 1.** Definitions and Coding of Sociodemographic Data Drawn From Electronic Health Record (EHR) **eTable 2.** Sociodemographic Characteristics for Patients Seen in Pre-implementation and Implementation Periods (N=409,217) **eTable 3.** Sleep Disorder Diagnosis Prevalence During Pre-implementation and Network-Wide Implementation Periods (N=409,217) **eTable 4.** Original Sleep Screener Item Wording by Age During Implementation Period **eFigure.** Rates of Overall and Practice-Level Adoption in Implementation and Maintenance Periods

Supplement 2.
Data Sharing Statement

## References

[1] Matricciani L, Paquet C, Galland B, Short M, Olds T. Children’s sleep and health: a meta-review. Sleep Med Rev. 2019;46:136-150. doi:10.1016/j.smrv.2019.04.011 31121414

[2] Dewald JF, Meijer AM, Oort FJ, Kerkhof GA, Bögels SM. The influence of sleep quality, sleep duration and sleepiness on school performance in children and adolescents: a meta-analytic review. Sleep Med Rev. 2010;14(3):179-189. doi:10.1016/j.smrv.2009.10.004 20093054

[3] Mietchen JJ, Bennett DP, Huff T, Hedges DW, Gale SD. Executive function in pediatric sleep-disordered breathing: a meta-analysis. J Int Neuropsychol Soc. 2016;22(8):839-850. doi:10.1017/S1355617716000643 27481012

[4] Fernandes SN, Zuckerman E, Miranda R, Baroni A. When night falls fast: sleep and suicidal behavior among adolescents and young adults. Child Adolesc Psychiatr Clin N Am. 2021;30(1):269-282. doi:10.1016/j.chc.2020.08.009 33223066 PMC7685287

[5] Honaker SM, Meltzer LJ. Sleep in pediatric primary care: a review of the literature. Sleep Med Rev. 2016;25:31-39. doi:10.1016/j.smrv.2015.01.004 26163054

[6] Hagan JF, Shaw J, Duncan P. Bright Futures. American Academy of Pediatrics Itasca; 2017.

[7] Marcus CL, Brooks LJ, Draper KA, ; American Academy of Pediatrics. Diagnosis and management of childhood obstructive sleep apnea syndrome. Pediatrics. 2012;130(3):576-584. doi:10.1542/peds.2012-1671 22926173

[8] Owens JA, Dalzell V. Use of the ‘BEARS’ sleep screening tool in a pediatric residents’ continuity clinic: a pilot study. Sleep Med. 2005;6(1):63-69. doi:10.1016/j.sleep.2004.07.015 15680298

[9] Honaker SM, McQuillan ME, Mindell JA, Downs SM, Slaven JE, Schwichtenberg AJ. Screening for problematic sleep in a diverse sample of infants. J Pediatr Psychol. 2021;46(7):824-834. doi:10.1093/jpepsy/jsab050 34283243 PMC8357224

[10] Honaker SM, Street A, Daftary AS, Downs SM. The use of computer decision support for pediatric obstructive sleep apnea detection in primary care. J Clin Sleep Med. 2019;15(3):453-462. doi:10.5664/jcsm.7674 30853049 PMC6411194

[11] Glasgow RE, Askew S, Purcell P, . Use of RE-AIM to address health inequities: application in a low-income community health center based weight loss and hypertension self-management program. Transl Behav Med. 2013;3(2):200-210. doi:10.1007/s13142-013-0201-8 23750180 PMC3671594

[12] Glasgow RE, Harden SM, Gaglio B, . RE-AIM planning and evaluation framework: adapting to new science and practice with a 20-year review. Front Public Health. 2019;7:64. doi:10.3389/fpubh.2019.00064 30984733 PMC6450067

[13] Moon RY, Darnall RA, Feldman-Winter L, Goodstein MH, Hauck FR; Task Force on Sudden Infant Death Syndrome. SIDS and other sleep-related infant deaths: updated 2016 recommendations for a safe infant sleeping environment. Pediatrics. 2016;138(5). doi:10.1542/peds.2016-2940 27940804

[14] Bombard JM, Kortsmit K, Warner L, . Vital signs: trends and disparities in infant safe sleep practices—United States, 2009–2015. MMWR Morb Mortal Wkly Rep. 2018;67(1):39-46. doi:10.15585/mmwr.mm6701e1 29324729 PMC5769799

[15] Whiteside-Mansell L, Nabaweesi R, Caballero AR, Mullins SH, Miller BK, Aitken ME. Assessment of safe sleep: validation of the parent newborn sleep safety survey. J Pediatr Nurs. 2017;35:30-35. doi:10.1016/j.pedn.2017.02.033 28728766 PMC5592828

[16] Chervin RD, Weatherly RA, Garetz SL, . Pediatric sleep questionnaire: prediction of sleep apnea and outcomes. Arch Otolaryngol Head Neck Surg. 2007;133(3):216-222. doi:10.1001/archotol.133.3.216 17372077

[17] Williamson AA, Mindell JA, Hiscock H, Quach J. Child sleep behaviors and sleep problems from infancy to school-age. Sleep Med. 2019;63:5-8. doi:10.1016/j.sleep.2019.05.003 31600659 PMC6859188

[18] Van Dyk TR, Becker SP, Byars KC. Mental health diagnoses and symptoms in preschool and school age youth presenting to insomnia evaluation: prevalence and associations with sleep disruption. Behav Sleep Med. 2019;17(6):790-803. doi:10.1080/15402002.2018.1518224 30260686 PMC6526081

[19] Adavadkar PA, Pappalardo AA, Glassgow AE, . Rates of diagnoses of sleep disorders in children with chronic medical conditions. J Clin Sleep Med. 2022;18(8):2001-2007. doi:10.5664/jcsm.10064 35621126 PMC9340607

[20] American Academy of Sleep Medicine. *International Classification of Sleep Disorders, Third Edition–Text Revision*. American Academy of Sleep Medicine; 2023.

[21] Quach J, Hiscock H, Ukoumunne OC, Wake M. A brief sleep intervention improves outcomes in the school entry year: a randomized controlled trial. Pediatrics. 2011;128(4):692-701. doi:10.1542/peds.2011-0409 21890825

[22] Mindell JA, Owens JA. A Clinical Guide to Pediatric Sleep: Diagnosis and Management of Sleep Problems. Lippincott Williams & Wilkins; 2015.

[23] Sadeh A. A brief screening questionnaire for infant sleep problems: validation and findings for an Internet sample. Pediatrics. 2004;113(6):e570-e577. doi:10.1542/peds.113.6.e570 15173539

[24] Forrest CB, Meltzer LJ, Marcus CL, . Development and validation of the PROMIS pediatric sleep disturbance and sleep-related impairment item banks. Sleep. 2018;41(6):zsy054. doi:10.1093/sleep/zsy054 29546286

[25] Meltzer LJ, Williamson AA, Mindell JA. Pediatric sleep health: it matters, and so does how we define it. Sleep Med Rev. 2021;57:101425. doi:10.1016/j.smrv.2021.101425 33601324 PMC9067252

[26] Paruthi S, Brooks LJ, D’Ambrosio C, . Recommended amount of sleep for pediatric populations: a consensus statement of the American Academy of Sleep Medicine. J Clin Sleep Med. 2016;12(6):785-786. doi:10.5664/jcsm.5866 27250809 PMC4877308

[27] Wheaton AG, Jones SE, Cooper AC, Croft JB. Short sleep duration among middle school and high school students—United States, 2015. MMWR Morb Mortal Wkly Rep. 2018;67(3):85-90. doi:10.15585/mmwr.mm6703a1 29370154 PMC5812312

[28] Buxton OM, Chang AM, Spilsbury JC, Bos T, Emsellem H, Knutson KL. Sleep in the modern family: protective family routines for child and adolescent sleep. Sleep Health. 2015;1(1):15-27. doi:10.1016/j.sleh.2014.12.002 26779564 PMC4712736

[29] Owens JA, Spirito A, McGuinn M. The Children’s Sleep Habits Questionnaire (CSHQ): psychometric properties of a survey instrument for school-aged children. Sleep. 2000;23(8):1043-1051. doi:10.1093/sleep/23.8.1d 11145319

[30] Adolescent Sleep Working Group; Committee on Adolescence; Council on School Health. School start times for adolescents. Pediatrics. 2014;134(3):642-649. doi:10.1542/peds.2014-1697 25156998 PMC8194457

[31] Liu Y, Zhang J, Li SX, . Excessive daytime sleepiness among children and adolescents: prevalence, correlates, and pubertal effects. Sleep Med. 2019;53:1-8. doi:10.1016/j.sleep.2018.08.028 30384136

[32] Janssen KC, Phillipson S, O’Connor J, Johns MW. Validation of the Epworth Sleepiness Scale for children and adolescents using Rasch analysis. Sleep Med. 2017;33:30-35. doi:10.1016/j.sleep.2017.01.014 28449902

[33] Allen SL, Howlett MD, Coulombe JA, Corkum PV. ABCs of SLEEPING: a review of the evidence behind pediatric sleep practice recommendations. Sleep Med Rev. 2016;29:1-14. doi:10.1016/j.smrv.2015.08.006 26551999

[34] Trinkley KE, Kroehl ME, Kahn MG, . Applying clinical decision support design best practices with the practical robust implementation and sustainability model versus reliance on commercially available clinical decision support tools: randomized controlled trial. JMIR Med Inform. 2021;9(3):e24359. doi:10.2196/24359 33749610 PMC8077777

[35] Jackson CL, Walker JR, Brown MK, Das R, Jones NL. A workshop report on the causes and consequences of sleep health disparities. Sleep. 2020;43(8):zsaa037. doi:10.1093/sleep/zsaa037 32154560 PMC7420527

[36] Fernandez-Mendoza J, Bourchtein E, Calhoun S, . Natural history of insomnia symptoms in the transition from childhood to adolescence: population rates, health disparities, and risk factors. Sleep. 2021;44(3):zsaa187. doi:10.1093/sleep/zsaa187 32929504 PMC7953218

[37] Wang Y, Zhao Z, Zhang Y, . Race, ethnicity, and sleep in US children. JAMA Netw Open. 2024;7(12):e2449861. doi:10.1001/jamanetworkopen.2024.49861 39656455 PMC11632548

[38] Flanagin A, Frey T, Christiansen SL; AMA Manual of Style Committee. Updated guidance on the reporting of race and ethnicity in medical and science journals. JAMA. 2021;326(7):621-627. doi:10.1001/jama.2021.13304 34402850

[39] Noelke C, McArdle N, Baek M, . Childhood opportunity index 2.0: technical documentation. Brandeis University. January 15, 2020. Accessed May 4, 2024. https://www.diversitydatakids.org/sites/default/files/2020-02/ddk_coi2.0_technical_documentation_20200212.pdf

[40] Pediatric Research Consortium. CHOP care network information for grants: 2023-24. Children’s Hospital of Philadelphia. Accessed May 1, 2024. https://www.research.chop.edu/sites/default/files/web/sites/default/files/pdfs/PeRC_CHOP_Care_Network_Info_Grants_2023-24.pdf

[41] Akoglu H. User’s guide to correlation coefficients. Turk J Emerg Med. 2018;18(3):91-93. doi:10.1016/j.tjem.2018.08.001 30191186 PMC6107969

[42] Archbold KH, Pituch KJ, Panahi P, Chervin RD. Symptoms of sleep disturbances among children at two general pediatric clinics. J Pediatr. 2002;140(1):97-102. doi:10.1067/mpd.2002.119990 11815771

[43] Meltzer LJ, Plaufcan MR, Thomas JH, Mindell JA. Sleep problems and sleep disorders in pediatric primary care: treatment recommendations, persistence, and health care utilization. J Clin Sleep Med. 2014;10(4):421-426. doi:10.5664/jcsm.3620 24733988 PMC3960385

[44] Anand V, Carroll AE, Downs SM. Automated primary care screening in pediatric waiting rooms. Pediatrics. 2012;129(5):e1275-e1281. doi:10.1542/peds.2011-2875 22508925 PMC3340595

[45] Carroll AE, Bauer NS, Dugan TM, Anand V, Saha C, Downs SM. Use of a computerized decision aid for developmental surveillance and screening: a randomized clinical trial. JAMA Pediatr. 2014;168(9):815-821. doi:10.1001/jamapediatrics.2014.464 25022724 PMC10157652

[46] Jenssen BP, DiFiore G, Powell M, . Accelerating innovation in primary care to support adolescent health discussions. Pediatrics. 2024;154(1):e2023064285. doi:10.1542/peds.2023-064285 38836314

[47] Lyon AR, Koerner K. User-centered design for psychosocial intervention development and implementation. Clin Psychol (New York). 2016;23(2):180-200. doi:10.1111/cpsp.12154 29456295 PMC5812700

[48] Mindell JA, Bartle A, Ahn Y, . Sleep education in pediatric residency programs: a cross-cultural look. BMC Res Notes. 2013;6(1):130. doi:10.1186/1756-0500-6-130 23552445 PMC3621514

[49] Calhoun SL, Fernandez-Mendoza J, Vgontzas AN, Liao D, Bixler EO. Prevalence of insomnia symptoms in a general population sample of young children and preadolescents: gender effects. Sleep Med. 2014;15(1):91-95. doi:10.1016/j.sleep.2013.08.787 24333223 PMC3912735

[50] Racine N, McArthur BA, Cooke JE, Eirich R, Zhu J, Madigan S. Global prevalence of depressive and anxiety symptoms in children and adolescents during COVID-19: a meta-analysis. JAMA Pediatr. 2021;175(11):1142-1150. doi:10.1001/jamapediatrics.2021.2482 34369987 PMC8353576

[51] Mahoney P, Solomon BS, McDonald EM, Shields WC, Gielen AC. Disclosure of infant unsafe sleep practices by African American mothers in primary care settings. JAMA Pediatr. 2019;173(9):878-879. doi:10.1001/jamapediatrics.2019.1687 31260002 PMC6604098

