# Supplementary material for: Implementing an Electronic Health Record–Integrated Pediatric Primary Care Sleep Screener
Source: JAMA Netw Open. 2025 Aug 5;8(8):e2525346. doi: 10.1001/jamanetworkopen.2025.25346 (PMC12556643; doi:10.1001/jamanetworkopen.2025.25346)
Supplement: Supplement 1. — eTable 1. Definitions and Coding of Sociodemographic Data Drawn From Electronic Health Record (EHR) eTable 2. Sociodemographic Characteristics for Patients Seen in Pre-implementation and Implementation Periods (N=409,217) eTable 3. Sleep Disorder Diagnosis Prevalence During Pre-implementation and Network-Wide Implementation Periods (N=409,217) eTable 4. Original Sleep Screener Item Wording by Age During Implementation Period eFigure. Rates of Overall and Practice-Level Adoption in Implementation and Maintenance Periods [file jamanetwopen-e2525346-s001.pdf]

Supplemental Online Content

Williamson AA, Powell M, Luberti A, et al. Implementing an electronic health record–integrated pediatric primary care sleep screener. *JAMA Netw Open*. 2025;8(8):e2525346. doi:10.1001/jamanetworkopen.2025.25346

eTable 1. Definitions and Coding of Sociodemographic Data Drawn From Electronic Health Record (EHR)

eTable 2. Sociodemographic Characteristics for Patients Seen in Pre-implementation and Implementation Periods (n=409,217)

eTable 3. Sleep Disorder Diagnosis Prevalence During Pre-implementation and Network-Wide Implementation Periods (n=409,217)

eTable 4. Original Sleep Screener Item Wording by Age During Implementation Period

eFigure. Rates of Overall and Practice-Level Adoption in Implementation and Maintenance Periods

This supplemental material has been provided by the authors to give readers additional information about their work.

© 2025 Williamson AA et al. *JAMA Network Open.*

eTable 1. Definitions and coding of sociodemographic data drawn from electronic health record (EHR)

Variable	Definition	Coding as covariate in analysis
Child age	• Generated from date of birth and date of patient's first well visit during data extraction period (i.e., pre-implementation or implementation year 1)	• Categorized into groups to align with age-based screener items (0-5 months, 6-11 months, 12-23 months, 24-35 months, 3-5 years, 6-12 years, 13+ years)
Child sex	• Sex assigned at birth entered by patient/family or staff member upon patient registration	• Dichotomized with male=1
Child race and ethnicity	• Race and ethnicity entered by patient/family or staff member upon patient registration into hospital-defined categories.<br>• Race and ethnicity are socio-political constructs and not indicators of genetic or biological factors	• The "other" category reflects patient/family-selected "other" race and/or racial groups with very small sample sizes; this category was collapsed with "multiple races" in analyses<br>• Non-Hispanic/Latine White race was selected as the reference group in analyses to reflect racial privilege at multiple systems levels, including in healthcare contexts.
Child insurance	• Medicaid/public, private, or self-pay insurance entered by patient/family or staff member	• Given low data coverage, self-pay was collapsed with private insurance for analyses to create a dichotomized variable (Private/self-pay =1)
Childhood Opportunity Index	• Geocoded residential addresses entered by patient/family and linked to Childhood Opportunity Index values<br>• Reflects 29 Census tract-level neighborhood educational (e.g., high school graduation rate), health and environmental (e.g., access to green space), and social (e.g., median household income) that yield a total score (range = 1-100), with higher scores suggesting greater advantage	• Categorized according to published Childhood Opportunity Index guidance: very low (score=1-20), low (21-40), moderate (41-60), high (61-80), and very high (81-100).

© 2025 Williamson AA et al. *JAMA Network Open.*

eTable 2. Sociodemographic characteristics for patients seen in pre-implementation and implementation periods (n=409,217)

Sociodemographic characteristics	Pre-implementation n=204,345		Implementation n=204,872			
	n	%	n	%	p-value	φ/Cramer's V
Age groups						
0-5 months	19307	9.5	16730	8.2	<0.001	0.039
6-11 months	6655	3.3	5476	2.7		
12-23 months	13005	6.4	11604	5.7		
24-35 months	11303	5.5	11751	5.7		
3-5 years	35275	17.3	34699	16.9		
6-12 years	72786	35.6	73951	36.1		
13+ years	46014	22.5	50661	24.7		
[a]Sex						
Female	99820	48.8	99890	48.8	0.56	0.0009
Male	104520	51.2	104972	51.2		
[a]Race and ethnicity					<0.001	0.033
African American/Black	9854	4.8	10675	5.2		
Asian	50523	24.8	46601	22.9		
Hispanic/Latine	17004	8.4	18776	9.2		
Multiple races	5604	2.8	7115	3.5		
Other	18498	9.1	18042	8.9		
White, non-Hispanic/Latine	101935	50.1	102344	50.3		
[a]Insurance						
Medicaid	59725	29.5	61156	30.0	<0.001	0.006
Private insurance	143021	70.5	142744	70.0		
Self-pay	8	0.0	5	0.0		
[a]COI						
Very low	36382	19.1	32592	17.1	<0.001	0.034
Low	28184	14.8	26767	14.0		
Moderate	31732	16.7	31178	16.4		
High	39955	21.0	41507	21.8		
Very High	54319	28.5	58590	30.7		

Note. COI = childhood opportunity index. Pre-implemetation =11/1/2018- 9/30/2019; implementation = 7/1/2021- 6/30/2022. N= 120,910 patients were seen in both periods. While associations were statistically significant in chi-squared tests, Phi and Cramer's V indicated negligible differences (< 0.05). [a]Unknown data for race or ethnicity (n=2,246, 0.5%), sex (n=15, 0.004%), insurance (n=2,558, 0.6%), and COI (n=28,011, 6.8%).

© 2025 Williamson AA et al. *JAMA Network Open.*

eTable 3. Sleep disorder diagnosis prevalence during pre-implementation and network-wide implementation periods (n=409,217)

Sleep disorder diagnosis	Pre-implementation (11/1/2018-9/30/2019) n=204,345		Network-wide implementation (7/1/2021-6/30/2022) n=204,872	
	n	%	n	%
Any diagnosis	3446	1.7	5188	2.5
Sleep disordered breathing	1989	1.0	2739	1.3
Unspecified sleep disturbance	1154	0.6	1992	1.0
Parasomnias	100	0.05	137	0.1
Inadequate sleep hygiene	68	0.03	132	0.1
Insomnia disorder	42	0.02	92	0.04
Insufficient sleep	31	0.02	20	0.01
Circadian rhythm sleep-wake disorders	24	0.01	25	0.01
Narcolepsy or hypersomnia disorders	17	0.01	22	0.01
Nightmares	11	0.01	14	0.01
Sleep-related movement disorders	8	<0.01	14	0.01
Sleep myoclonus	2	<0.01	1	<0.01

Note. N=120,910 patients were seen in both periods. Sleep disorder diagnoses were identified using International Classification of Diseases (10th edition) codes and classified into broader categories consistent with prior research, as follows: Sleep disordered breathin = R06.81, R06.83, G47.3, G47.30, G47.31, G47.33, G47.34, G47.36, G47.37, and G47.39; unspecific sleep disturbance= G47.8 and G47.9; parasomnias (non-Rapid Eye Movement)= F51.3, F51.4, G47.5, G47.50, G47.51, G47.54, and G47.59; inadequate sleep hygiene= Z72.820; insomnia disorder= A81.83; F51.01, F51.02, F51.03, F51.04, F51.05, F51.09, G47.00, G47.01, G47.09, Z73.81, Z73.810, Z73.811, Z73.812, and Z73.819; insufficient sleep=F51.12 and Z72.820; circadian rhythm sleep-wake disorders= G47.2, G47.20, G47.21, G47.22, G47.23, G47.24, G47.25, G47.26, G47.27, and G47.29; narcolepsy or hypersomnia disorders= G47.1, G47.10, G47.11, G47.12, G47.13, G47.14, G47.19, G47.4, G47.41, G47.411, G47.419, G47.42, G47.421, and G47.429; nightmares= F51.5; sleep-related movement disorders= G47.6, G47.61, G47.62, G47.63, and G47.81; sleep myoclonus= G25.3.

© 2025 Williamson AA et al. *JAMA Network Open.*

eTable 4. Original sleep screener item wording by age during implementation period

Key sleep domain	Question wording by age
Sleep position	• **0-5 months and 6-11 months:** Does your baby sleep with you or another adult?<br>• **All other age groups:** Not included due to older age.
Sleep disordered breathing	• **0-5 months:** Not included due to young age.<br>• **6-11 months:** Does your baby snore 3 or more nights per week (when not sick)?<br>• **12-23 months, 24-35 months, and 3-5 years:** Does your child snore 3 or more nights per week (when not sick)?<br>• **6-12 years:** Does your child/do you snore 3 or more nights per week (when not sick)?<br>• **13+ years:** Does your teen/do you snore 3 or more nights per week (when not sick)?
Perceived sleep problem	• **0-5 months:** Not included due to young age and normative night awakenings in infancy.[30]<br>• **6-11 months:** Are there any problems with your baby's sleep?<br>• **12-23 months, 24-35 months, and 3-5 years:** Are there any problems with your child's sleep?<br>• **6-12 years:** Are there any problems with your child's/your sleep?<br>• **13+ years:** Are there any problems with your teen's/your sleep?
Insufficient sleep	• **12-23 months:** Does your child usually get less than 11 hours of sleep per day, including naps?<br>• **3-5 years:** Does your child usually get less than 10 hours of sleep per day on school/weeknights, including naps?<br>• **6-12 years:** Does your child/do you usually get less than 9 hours of sleep per day on school/weeknights?<br>• **13+ years:** Does your teen/do you usually get less than 8 hours of sleep per day on school/weeknights?
Daytime sleepiness	• **All younger ages:** Not included due to young age and/or need for screener brevity.<br>• **13+ years:** Does your teen/do you ever fall asleep in school?

Note. Sources for question wording shown in manuscript Box 1.Well visits by age: 0-5 months= newborn and 1, 2, and 4-month visits; 6-11 months= 6 and 9-month visits; 12-23 months= 12, 15, and 18-month visits; 24-35 months= 24 and 30-month visits; 3-5 years= 3-year, 42-month, 4-year, and 5-year visits; 6-12 years = 6, 7, 8, 9, 10, 11, 12-year visits; 13+ years= 13, 14, 15, 16, 17, 18-year visits. Implementation = 7/1/2021- 6/30/2022.

© 2025 Williamson AA et al. *JAMA Network Open.*

eFigure. Rates of overall and practice-level adoption in implementation and maintenance periods

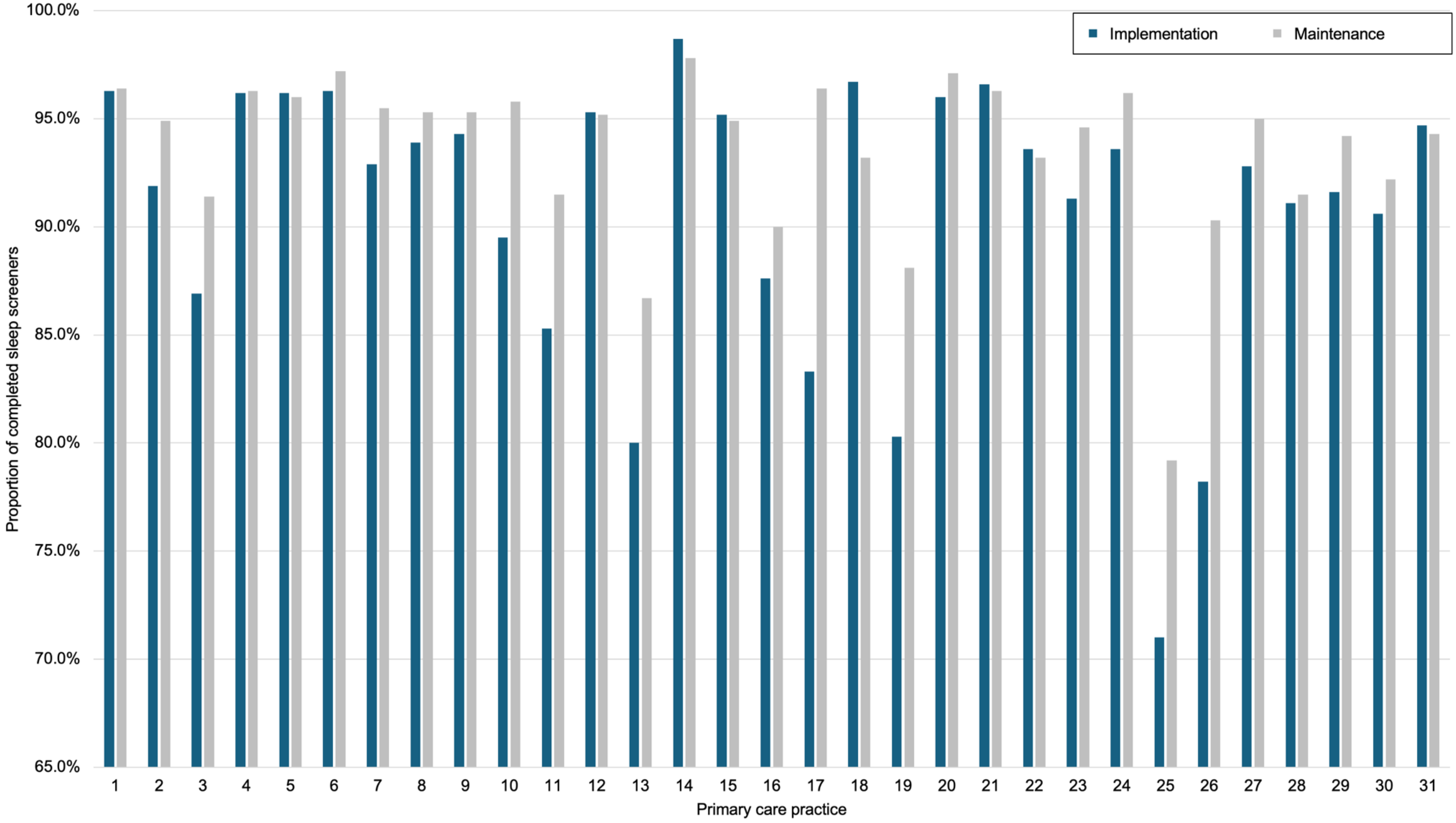

Figure Legend: Rates of adoption at each of the 31 practices during the implementation (07/01/2021-06/30-2022) and maintenance (07/01/2022-07/01/2023) periods. Adoption rates were calculated by dividing the number of completed well visit sleep screeners by the number of completed well visit encounters during each period. A rate of 80% or above is considered to be high adoption.

© 2025 Williamson AA et al. *JAMA Network Open.*
