# Supplementary material for: Implementing an Electronic Health Record–Integrated Pediatric Primary Care Sleep Screener
Source: JAMA Netw Open. 2025 Aug 5;8(8):e2525346. doi: 10.1001/jamanetworkopen.2025.25346 (PMC12556643; doi:10.1001/jamanetworkopen.2025.25346)
Supplement: Supplement 2. — Data Sharing Statement [file jamanetwopen-e2525346-s002.pdf]

Data Sharing Statement

Williamson. Implementing an Electronic Health Record–Integrated Pediatric Primary Care Sleep Screener. *JAMA Netw Open*. Published August 05, 2025. doi:10.1001/jamanetworkopen.2025.25346

Data
Data available: No

Additional Information
Explanation for why data not available: Deidentified individual participant data will be made available upon approval of outside requests by the study team and with appropriate human subjects permissions.
